# An Electroencephalography Profile of Paroxysmal Kinesigenic Dyskinesia

**DOI:** 10.1002/advs.202306321

**Published:** 2024-01-16

**Authors:** Huichun Luo, Xiaojun Huang, Ziyi Li, Wotu Tian, Kan Fang, Taotao Liu, Shige Wang, Beisha Tang, Ji Hu, Ti‐Fei Yuan, Li Cao

**Affiliations:** ^1^ Department of Neurology Shanghai Sixth People's Hospital Affiliated to Shanghai Jiao Tong University School of Medicine Shanghai 200233 China; ^2^ Shanghai Key Laboratory of Psychotic Disorders, Brain Health Institute, National Center for Mental Disorders, Shanghai Mental Health Center Shanghai Jiao Tong University School of Medicine Shanghai 200030 China; ^3^ Department of Neurology Shanghai General Hospital, Shanghai Jiao Tong University Shanghai China; ^4^ Department of Neurology Xiangya Hospital, Central South University Hunan Province 410008 China; ^5^ School of Life Science and Technology ShanghaiTech University Shanghai 201210 China; ^6^ Co‐innovation Center of Neuroregeneration Nantong University Nantong Jiangsu 226019 China; ^7^ Institute of Mental Health and drug discovery Oujiang Laboratory (Zhejiang Lab for Regenerative Medicine, Vision and Brain Health) Wenzhou Zhejiang 325000 China; ^8^ Shanghai Neurological Rare Disease Biobank and Precision Diagnostic Technical Service Platform Shanghai China

**Keywords:** functional connectivity, high‐density electroencephalogram, oscillatory activity, paroxysmal kinesigenic dyskinesia

## Abstract

Paroxysmal kinesigenic dyskinesia (PKD) is associated with a disturbance of neural circuit and network activities, while its neurophysiological characteristics have not been fully elucidated. This study utilized the high‐density electroencephalogram (hd‐EEG) signals to detect abnormal brain activity of PKD and provide a neural biomarker for its clinical diagnosis and PKD progression monitoring. The resting hd‐EEGs are recorded from two independent datasets and then source‐localized for measuring the oscillatory activities and function connectivity (FC) patterns of cortical and subcortical regions. The abnormal elevation of theta oscillation in wildly brain regions represents the most remarkable physiological feature for PKD and these changes returned to healthy control level in remission patients. Another remarkable feature of PKD is the decreased high‐gamma FCs in non‐remission patients. Subtype analyses report that increased theta oscillations may be related to the emotional factors of PKD, while the decreased high‐gamma FCs are related to the motor symptoms. Finally, the authors established connectome‐based predictive modelling and successfully identified the remission state in PKD patients in dataset 1 and dataset 2. The findings establish a clinically relevant electroencephalography profile of PKD and indicate that hd‐EEG can provide robust neural biomarkers to evaluate the prognosis of PKD.

## Graphical abstract

A clinically relevant electroencephalography profile features of paroxysmal kinesigenic dyskinesia (PKD) is established by high‐density electroencephalogram (hd‐EEG) signals. Subtype analyses show that increased theta oscillation is related to psychological factors, while the decreased high‐gamma functional connectivity is associated with motor symptoms. The following remission state prediction model indicated that hd‐EEG can provide robust neural biomarkers to evaluate the prognosis of PKD.

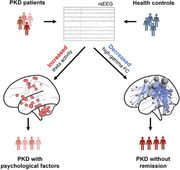

## 1 Introduction

Paroxysmal kinesigenic dyskinesia (PKD), characterized by transient and recurrent dystonic or choreoathetoid attacks precipitated by sudden movements, is the most common and representative type of paroxysmal dyskinesia.^[^ ^1^, ^2^ ^]^ The unique features of PKD include kinesigenic triggers, a tendency toward natural remission, and a dramatic response to epileptic drugs,^[^ ^3^ ^]^ suggesting distinct neural mechanisms from other movement disorders. Although several genetic factors for PKD have been identified since 2011,^[^ ^4^, ^5^, ^6^, ^7^ ^]^ the underlying mechanism of PKD is far from being fully elucidated.

Recently, structural and functional abnormalities involving the caudate nucleus, putamen, pallidum, thalamus, and frontoparietal cortex in PKD patients have been reported with different neuroimaging methods, including single‐photon emission computed tomography (SPECT) and positron emission tomography (PET). There were also alterations in interhemispheric functional connectivity between the brain regions of bilateral cerebral hemispheres, such as putamen, primary motor cortex, supplementary motor area, dorsal lateral prefrontal cortex, and primary somatosensory cortex.^[^ ^8^, ^9^ ^]^ In addition, cerebellar changes have been identified in Proline‐rich transmenbrane protein 2 (*PRRT2)*‐related PKD. In PKD patients with *PRRT2* mutations, it is presumed that abnormal cerebellar output is the primary dysfunction, which leads to striatal dysregulation and paroxysmal dyskinesia.^[^ ^10^ ^]^ In *PRRT2*‐deficient mice, dyskinetic movements were triggered due to the induction of cerebellar spreading.^[^ ^11^ ^]^ However, global characterization of PKD‐associated neural disruptions, especially the functional connectivity, is still lacking.^[^ ^12^, ^13^, ^14^, ^15^ ^]^

Electroencephalography (EEG) provides a clinically translatable assessment of whole‐brain neural activity at high temporal resolution. However, due to limited recording channels and the effects of volume conduction, conventional EEG could not precisely estimate the neuronal sources of the single brain region. By contrast, high‐density EEG (hd‐EEG) with 128 channels or higher offers a high spatial sampling of the potential distribution on the head with more information collected. Hd‐EEG can accurately depict the activity of a single brain region through the latest algorithms, including the subcortical nucleus and even the cerebellum, which had been confirmed by synchronously intracranial recording local field potentials of deep brain regions and hd‐EEG in a recent study.^[^ ^16^ ^]^ Furthermore, algorithms are constantly being designed to reduce the influence of volume conduction on source localization. For example, an algorithm for calculating functional connectivity called power envelope connectivity (PEC) solved this issue by removing signals with zero phase lag, which is the main reason for volume conduction, through the orthogonalizing amplitude of two signals. The neural oscillation activity patterns quantified by PEC were found related to some diseases,^[^ ^17^ ^]^ such as depression and PTSD, and can be used as neurobiomarkers to assist clinical diagnosis^[^ ^18^ ^]^ and disease prognosis.^[^ ^19^ ^]^

The current knowledge of the neural activity characteristics of PKD is only limited to scalp EEG, such as diffuse or focal slow waves and epileptic discharges.^[^ ^1^ ^]^ We aimed to discover the key brain region and whole brain dysfunction of PKD by taking advantage of hd‐EEG, high temporal and spatial resolution. To this end, both cortical and subcortical brain regions were evaluated by hd‐EEG source localization in this study. Oscillatory activities (PSD) of each region and functional connectivity (PEC) between paired regions were estimated and compared health controls (HCs) and PKD or subtypes of PKD (remission or non‐remission subtype, trigger factors: “puremove” or “non‐puremove” subtype). Finally, connectome‐based predictive modeling (CPM) for identifying remission PKD patients was established and validated by another group of independent data.

## 2 Experimental Section

### 2.1 Participants

**Figure 1:**
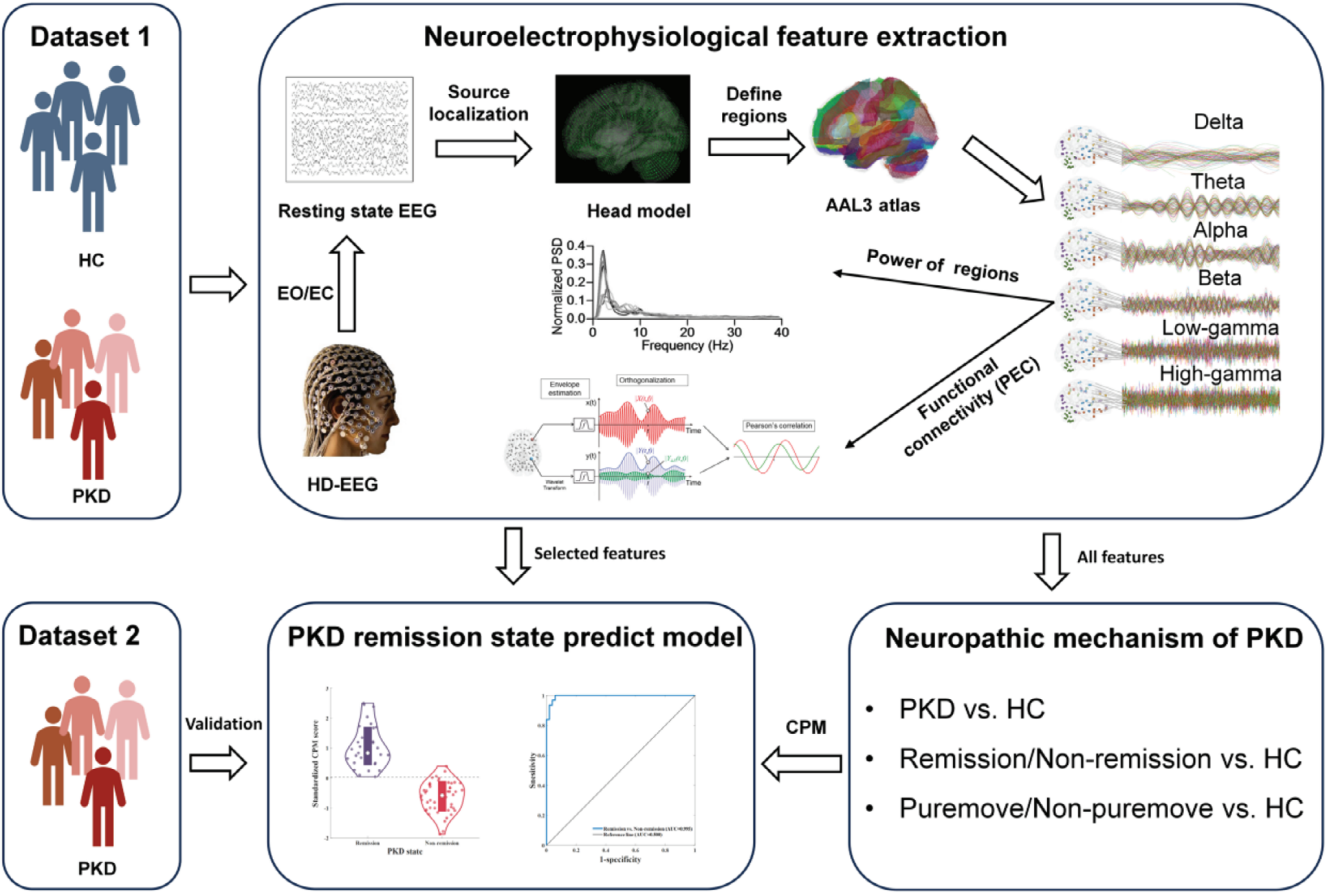
Overview of the design of research and main steps of analysis. Two independent EEG datasets were included in this research, dataset 1 was used for uncovering the neuropathic mechanism of PKD and training a prediction model for the classification of different PKD remission states, while dataset 2 was used as testing data for validation of the CPM. High‐density EEG was recorded under eye‐open (EO) and eye‐close (EC) paradigms. The activity of cortical and subcortical regions was estimated by source localization. Features of regions’ power functional connectivity (PEC) between two regions in six oscillations were calculated. Neural activity patterns of PKD were investigated by comparing features between HC and PKD or HC with different PKD subtypes. A CPM model was established based on features selected from dataset 1 and validated by dataset 2.

Two independent hd‐EEG datasets were included in the study. A total of 115 PKD patients and 108 HCs were recruited in dataset 1 (recruitment time 202103–202201), and 35 PKD patients in dataset 2 (recruitment time 202207–202303). Dataset 2 was collected to validate the generalization of the remission state prediction model established based on dataset 1. The clinical diagnosis of PKD is based on Bruno's criteria and recently modified diagnostic recommendations.^[^ ^3^, ^20^ ^]^ The disease duration of all the enrolled patients was at least one year. All participants or their guardians signed written informed consent, and ethics approval was granted by the research ethics boards of the Shanghai Sixth People's Hospital (Approval No: 2021–219). A schematic overview of the main procedure is illustrated in **Figure** **1**.

### 2.2 Assessment

Basic information, including age, gender, handedness, and education year, was collected from all participants. Clinical manifestations of PKD, including onset age, age of remission, triggers, aura, attack forms, duration, frequency of attacks, and reaction to anticonvulsants, were recorded using the PKD registration form designed by the Department of Neurology, Shanghai Sixth People's Hospital. Since medication might affect neural activity in the brain,^[^ ^21^, ^22^ ^]^ the medication taken by all participants was also recorded. For the HCs, the participants who took medications that would affect neural activity, such as antihypertensive, anticonvulsants, and benzodiazepines, will be excluded. For the PKD group, antiepileptic drugs, such as carbamazepine, lamotrigine, oxcarbazepine, etc., were considered as routine drugs. Therefore, the medication taken 3 days before the EEG recording was recorded. Patients who did not take the medication 3 days before the experiment were defined as the medication off (med‐off) group and vice versa were considered as the medication on (med‐on) group.

### 2.3 Resting‐State EEG Acquisition

A 129‐channel system (Electrical Geodesics, Eugene, OR, USA; www.egi.com, sampling rate, 500 Hz; bandpass, 0.5‐500 Hz) was used for hd‐EEG acquisition with Cz as a reference (Electrical Geodesics). During the recording, participants were asked to sit comfortably and remain relaxed. They were then instructed to keep still, minimize blinks or eye movement, and fixate on a centrally presented cross during the eyes‐open paradigms. Resting‐state EEGs were recorded in both eyes‐open and eyes‐closed paradigms, each for 5 min. If the participant fell asleep, they should report to the constructor. All the participants were instructed to keep awake during the recording and asked to report if they fell asleep after the data collection. If drowsiness or sleep is reported, the corresponding data will be eliminated.

### 2.4 EEG Pre‐Processing

First, the EEG signal was offline 1 Hz high‐pass filter (FIR 1651 point high‐pass filter, transition bandwidth: 1 Hz, passband edge: 1 Hz, cutoff frequency (−6 dB): 0.5 Hz) to remove linear trends, notch filtered to remove 50 Hz line noise (FIR 1651 point notch filter, transition bandwidth: 1 Hz, passband edges: [49 51] Hz, cutoff frequencies (−6 dB), [49.5 50.5] Hz), 90 Hz low‐pass filter (FIR 75 point low‐pass filter, transition bandwidth: 22.5 Hz, passband edges: 90 Hz, cutoff frequency (−6 dB), 101.25 Hz). Second, topographically interpolated bad channels using spherical splines, and re‐referenced to the grand average. Third, ocular, muscular, and cardiac artifacts were then eliminated with independent component analysis (ICA). Fourth, the analyzed signals were segmented into 2‐s segments, and amplitudes >100 µV were excluded. All steps were conducted by using the EEGLAB toolbox (https://sccn.ucsd.edu/eeglab/index.php). After pre‐processing, subjects with >15% bad channels or >50% excluded data segments were not included in further analysis.

### 2.5 Power Spectrum and Functional Connectivity Analyses in Source Space

Source localization to the cortical and subcortical brain areas was accomplished using the Brainstorm toolbox (http://neuroimage.usc.edu/brainstorm). The minimum‐norm estimation approach was applied to convert the channel‐space EEG into the source‐space signals of 1922 vertices. Specifically, a three‐layer (scalp, skull, and cortical surface) boundary element head model was computed with the OpenMEEG plugin based on the FreeSurfer average brain template. A total of 16 088 dipoles with unconstrained orientations were generated. The lead‐field matrix relating the dipole activities to the EEG was obtained as a result of the boundary element modeling. Principal component analysis was employed to reduce the 3D estimated source signal at each voxel to the 1D time series of the principal component. Then, 149 brain regions were defined in the Montreal Neurological Institute (MNI) space based on the AAL3 template (https://www.gin.cnrs.fr/en/tools/aal/). For source localization reliability, brain regions with voxels less than five were excluded, and all the subregions of the thalamus were merged into one region. As a result, 113 regions were preserved and divided into nine functional groups (DMN: default mode network; DAN: dorsal attention network; SMN: somatomotor network; VAN: ventral attention network; FPN: frontoparietal network; VN: visual network; LN: limbic network; SC: subcortical regions; CB: cerebellum), which included seven cortical functional networks from the Buckner group,^[^ ^23^ ^]^ a subcortical region and the cerebellum regions.

Six canonical frequency band oscillations, delta (2–4 Hz), theta (5–7 Hz), alpha (8–12 Hz), beta (13–29 Hz), low‐gamma (30–59 Hz), and high‐gamma (60–90 Hz), were then extracted at each region via the wavelet transform with the complex Morlet wavelet (central frequency *fc* = 1 Hz, time resolution (FWHM_tc) = 3 s, the temporal resolution of frequency “f” is FWHM_tc * fc/f). The source‐space power spectrum density (PSD) was calculated using the windowed fast Fourier transform with a 2 s sliding window and 1 s overlap. To reduce the influence of inter‐subject variability, the power spectra were normalized with a z‐transform within 2–90 Hz. The activity level of each oscillation is the area under the PSD curve within its frequency band. The power envelope connectivity (PEC) was exploited to estimate brain functional connectivity (FC). PEC measures the correlation between the power envelopes of two band‐limited spontaneous brain signals and characterizes the amplitude synchrony between any pair of brain regions.^[^ ^24^ ^]^ By orthogonalizing the analytical time series of the two brain signals before calculating the power envelopes, PEC removes the zero‐phase‐lag connectivity, which is the primary source of spurious connectivity due to volume conduction (one of the main challenges to the analysis of FC using EEG). PEC was a reliable FC measure widely used in magnetoencephalography (MEG)^[^ ^25^ ^]^ and EEG.^[^ ^18^, ^26^ ^]^ The regional pairwise PEC features were further extracted among 113 regions, and 6441 unique regional pairwise connectivity features were computed in each of the six frequency band oscillations. The PEC features were finally normalized by z‐transform. More details about PEC can be found in the Supporting Information.

### 2.6 The Discrepancy in Functional Connectivity Between PKD Subtypes and HCs

Average PEC values between all paired regions were generated within the HC group for each oscillation. The difference in PEC between PKD patients and HCs at each oscillation was defined as the sum of the discrepancies in each functional connectivity.

### 2.7 Identification of Remission State Based on CPM

To assess PSD and PEC as potential biomarkers in distinguishing remission PKD patients from non‐remission, the conducted CPM^[^ ^26^ ^]^ in MATLAB with validated custom scripts (https://github.com/YaleMRRC/CPM). CPM generates a binary classification model with group labels (here, remission or non‐remission). Both PSD of essential regions and whole brain PEC with a significant difference were applied as inputs. The CPM identifies positive and negative predictive features in a training dataset using a dependent *t*‐test. A CPM score, the sum of all positive and negative features, is then calculated for each individual and entered into predictive models. Resultant classification thresholds were then applied to the test dataset to generate predictions, and model performance was quantified as the correspondence between actual and predicted labels. A schematic of CPM is supplied in Figure S2 (Supporting Information). To further validate the CPM's robustness for PKD patients' remission state, the same CPM was used for classifying the state of remission in patients from dataset 2.

### 2.8 Statistical Analysis

The student's *t*‐test was used to analyze PSD or PEC values of any region‐of‐interest pair in six oscillations in two groups. Permutation testing (permutation number is 1000) was performed to evaluate the statistical significance of all tests. *p* < 0.05 indicates a significant difference, and all reported *p* values were after FDR correction. Since the features' dimension far exceeded the classification dimension, the robustness of features are evaluated to avoid overfitting. In 100 replicates, 95% of each group were subsampled and the difference in PSD and PEC between the two groups was calculated. The final results only show the features with significant differences (*p* < 0.05). Candidate features for CPMs were selected based on their average performance in replicates. Accuracy and receiver operating characteristic (ROC) curve analyses were performed. The area under the curve (AUC) indicated the diagnostic ability of these variables.

## 3 Results

### 3.1 Epidemiologic Characteristics of Enrolled PKD Patients

After the data pre‐processing, 98 PKD patients and 96 HCs in dataset 1 and 31 PKD patients in dataset 2 were finally included. The reason for the exclusion of some subjects was due to the poor quality of electrophysiological data. Information about the number of bad channels, the ratio of bad epochs, and the number of epochs for analysis in PKD and HC groups were shown in Figure S3 (Supporting Information). In addition, there is no difference between HC and PKD in these three parameters of pre‐processed EEG data. Demographic variables are provided in **Table** **1**. There was no statistically significant difference between PKD patients and healthy controls in terms of gender, age, handedness, and education year. Detailed disease information of PKD patients is listed in **Table** **2**. Consistent with previous clinical data,^[^ ^1^ ^]^ the average onset years was 12.24 ± 3.36 years, and the average disease duration was 11.93 ± 8.19 years. A state of remission in PKD is defined as a reduction in the frequency or severity of attacks or complete absence of attack without the aid of any medications. In this study, 38 patients reported spontaneous remission, and the average age of remission was 21.26  ± 4.31 years. Five types of attack triggers/factors are classified into two categories. Motor triggers include sudden voluntary actions and action changes (changes in the speed or amplitude of movements), while physiologically aggravating factors include emotional factors, intent action, and fatigue. The ratio of patients triggered by action change, emotional factors, intent action, and fatigue was 77:21, 59:39, 59:39, and 33:65, respectively. The medication state of PKD patients when recording EEG: 64 patients were in a med‐off state, 26 were in a med‐on state, and eight were unknown.

**Table 1:** Basic information about participants of dataset 1 and dataset 2.

	Dataset 1: PKD [*n* = 98]		Dataset 1: HC [*n* = 96]		Dataset 2: PKD [*n* = 31]			
Gender	Male	Female	Male	Female	Male	Female	χ^2^	*p*
	77	21	74	22	26	2	2.629	0.269
Handedness	**Right**	**Left**	**Right**	**Left**	**Right**	**Left**		
	91	7	94	2	*31*	*0*	4.731	*0.094*
	**Mean**	**SD**	**Mean**	**SD**	**Mean**	**SD**	**F**	***p***
Age (years)	24.15	7.17	24.15	7.29	25.03	7.90	0.194	0.824
Education (years)	14.57	3.22	15.16	2.18	14.00	3.12	2.069	0.129

**Table 2:** Disease information of PKD patients from dataset 1 and dataset 2.

		Dataset 1			Dataset 2		
		Mean	SD		Mean	SD	
Onset age [year]		12.24	3.36		11.44	2.82	
Disease duration [year]		11.93	8.19		13.96	8.66	
		Yes	No	Unknown	Yes	No	Unknown
Medication Application		26	72	8	8	20	0
Relief of Motor Symptom		38	51	9	14	14	0
Types of Triggers	Sudden voluntary actions	98	0	0	27	0	1
	Change of Action	77	21	0	16	11	1
	Emotional Factors	59	39	0	17	10	1
	Intent action	59	39	0	12	15	1
	Fatigue	33	65	0	7	20	1

### 3.2 An Electroencephalography Profile of PKD

In order to have a comprehensive understanding of the electrophysiological profile of PKD, we analyzed resting‐state EEG data with eyes‐open (PKD *n* =  85, HC *n* = 85) and eyes‐close (PKD *n* = 86, HC = 88) paradigms. The abnormal neural activity patterns of PKD were highly consistent between the two paradigms, with only some differences in the specific brain regions involved. To highlight the altered neural activity patterns in PKD, we have placed the results for the eye‐open paradigm in Figures S4–S6 (Supporting Information). Previous studies^[^ ^28^, ^29^ ^]^ have suggested that under the eye‐close paradigm, external information input is reduced, and the participant can be in a better‐resting state and have less signal noise due to blinks, head movements, etc.

### 3.3 Abnormal Neural Activity in PKD

**Figure 2:**
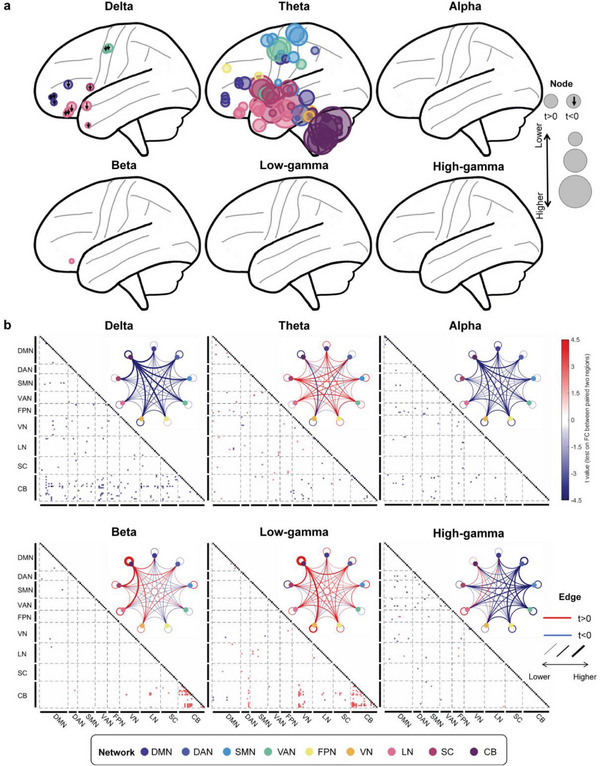
The discrepancy oscillatory activity pattern between PKD and HC on the eye‐close paradigm. a) Visualization of oscillatory activity difference (PKD vs HC) patterns on the brain. The node size at each significant region (corrected *p*‐value <0.05 after FDR correction) represents its absolute *t*‐value, the larger the size, the more significant it becomes. A downward arrow on the node indicates that the oscillatory activity of PKD is significantly lower than HC (*t*‐value <0), and the node without an arrow indicates that PKD is significantly higher than HC (*t*‐value > 0). b) Functional connectivity difference between PKD and HC. Squares in the triangular matrix are functional connectivity (FC) of paired brain regions, and only significant FCs are drawn (*p* < 0.05 after FDR correction). The black squares indicate that the diagonal elements in the FC matrices are non‐meaningful for our analysis. We averaged the *t*‐values for each network and displayed them with a circular plot. The circle color represents different networks; the lines between or within the circle are the averaged *t*‐value. The blue line indicates that the *t*‐value is >0, while the red line indicates that the *t*‐value is ˂0, and the thicker the line, the greater the absolute value of *t*. We divided the brain regions into nine groups according to their functional network membership. DMN: default mode network; DAN: Dorsal attention network; SMN: somatomotor network; VAN: ventral attention network; FPN: frontoparietal network; VN: visual network; LN: limbic network; SC: subcortical regions; CB: cerebellum.

Regarding the oscillatory activity of regions (PSD), the most significant difference between PKD and healthy controls (HC) appeared at theta oscillation. At the same time, discrepancies also existed in delta or beta oscillation (**Figure** **2**a; Figure S4a, Supporting Information). The difference was prominent both in eye‐open and eye‐closed paradigms. Compared with HC, PKD patients' activity at theta oscillation showed a widespread increase in all networks, especially in the cerebellum, subcortical, LN, SMN, and DMN. At the same time, an additional discrepancy was also found at the VN during the eye‐open paradigm. Moreover, activity at delta oscillation decreased in 11 regions (mainly from LN) in the eye‐closed paradigm, while the activity in three regions markedly decreased at delta oscillation in the eye‐open paradigm (IPL, postcentral, supramarginal). For beta oscillations, significant differences were identified only in the postcentral cortex and OFC in the eye‐open and eye‐closed paradigms, respectively.

The diversity at delta, low‐gamma, and beta FCs in the eye‐closed paradigm was noticeable between PKD patients and HCs (Figure 2b), and they were mainly associated with the cerebellum. Specifically, compared to HCs, the connection between the cerebellum and other brain regions at delta oscillation was weakened in PKD patients. In contrast, connectivity within the cerebellum at beta and low‐gamma oscillation was strengthened. However, only theta FCs were the most prominent difference in the eye‐open paradigm (Figure S4b, Supporting Information).

### 3.4 Neural Activity Changed After PKD Remission

**Figure 3:**
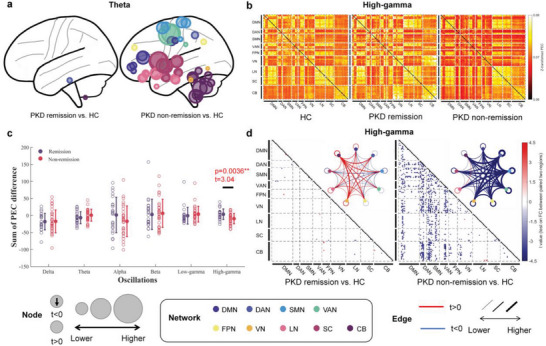
The discrepancy oscillatory activity pattern of remission and non‐remission PKD patients on the eye‐close paradigm. a) Regions with significantly increased theta activity between remission or non‐remission PKD patients and HCs. b) Mean high‐gamma oscillation FC matrices of all HCs, remission, and non‐remission PKD patients. c) The sum of PEC differences between remission or non‐remission PKD patients and HCs. The non‐remission PKD patients were significantly higher than the remission PKD patients at the PEC on high‐gamma oscillation. d) High‐gamma oscillation FC difference between remission or non‐remission PKD patients with HC. The black squares in (d) indicate that the diagonal elements in the FC matrices are non‐meaningful for our analysis.

Spontaneous remission is the distinctive characteristic of PKD. To explore the mechanism behind this phenomenon, we further investigated the difference in PSD and PEC features between PKD patients with different disease stages (non‐remission/remission) and HCs. To remove the effect of medication use on the results, we excluded those PKD patients who were in a med‐on state (eye‐open: non‐remission *n* = 38, remission *n* = 25; eye‐close: non‐remission *n* = 32, remission *n* = 23). In terms of PSD, non‐remission PKD patients showed notably decreased activity at theta oscillation in many regions compared with HCs (**Figure** **3a**; Figure S5a, Supporting Information), which is consistent with the findings in comparison between overall PKD and HCs, while only a few regions from right hemisphere with discrepancies were found in remission PKD patients (right middle frontal gyrus, Fusiform, inferior temporal gyrus, middle temporal gyrus, superior temporal gyrus, on eye‐open paradigm and right inferior temporal gurus and Lobule VIIB of the cerebellar hemisphere on eye‐closed paradigm). Regarding FC, a significant difference between the remission and non‐remission PKD patients was only found in high‐gamma FCs (Figure 3c; Figure S5c, Supporting Information) in both eye‐open (*t* = 3.01, *p* = 0.0037) and eye‐closed (*t* = 3.04, *p* = 0.0036) paradigms. As shown in Figure 3b and Figure S5b (Supporting Information), the broader range of connections was in non‐remission PKD patients than in HCs and remission PKD patients. Discrepant high‐gamma FCs between non‐remission PKD patients and HCs were mainly between the DMN, DAN, SMN, VAN, and other groups or within the DAN, SMN, VAN (Figure 3d; Figure S5d, Supporting Information). However, decreased FCs were not found in remission PKD patients. By contrast, some of them were even excessively enhanced.

### 3.5 The Specific Role of Theta and High‐Gamma Oscillations in PKD

Since we identified the notable involvement of theta oscillatory activity and high‐gamma FCs, we attempted to explore their specific roles in PKD further. The current definition of remission in PKD mainly refers to decreased attacks or relief of motor symptoms. At the same time, emotional factors have been identified to participate in PKD, acting as an additional trigger for attacks and aggravating factors for motor symptoms.^[^ ^19^ ^]^ In clinical practice, most patients reported anxiety and depression when they had symptoms.^[^ ^27^ ^]^ Based on these evidence, we hypothesize that theta and high‐gamma oscillations may play different roles in coding motor and emotional symptoms in PKD patients.

**Figure 4:**
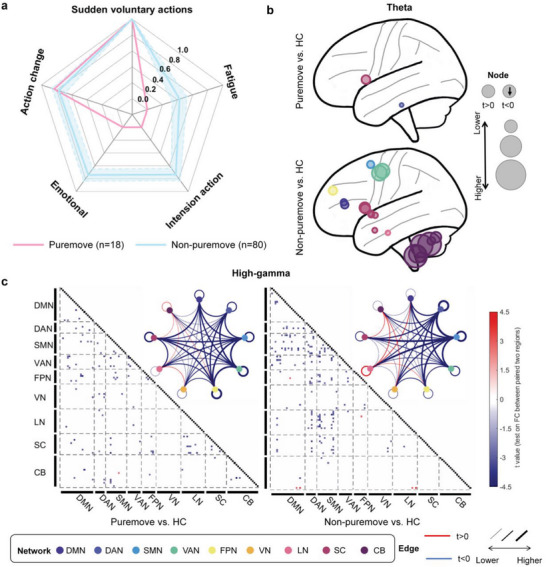
The discrepancy oscillatory activity pattern of two subtypes of PKD: “Puremove” and “Non‐puremove” on the eye‐close paradigm. a) The average of the five common triggering/factors may trigger a PKD attack in “puremove” and “non‐puremove” subtypes of all PKD patients. Blue shading is the standard deviation obtained for a random sample of 18 subjects in the non‐puremove group. b) Regions with significantly increased theta activity between the two subtypes and HC. c) High‐gamma oscillation FC difference between the two subtypes of PKD and HC.

Therefore, we divided PKD patients into two subtypes according to different triggers to verify this hypothesis. Among the five triggers of a PKD attack, “sudden voluntary actions” and “speed change” are more related to movement. In comparison, the other three, “emotional factors”, “intention action”, and “fatigue”, are aggravating factors and associated with psychological status. Subsequently, we divided PKD patients into the “puremove” subtype, which only has at least one movement trigger, and the “non‐puremove” subtype, which triggers should contain both movement triggers and psychologically aggravating factors (**Figure** **4a**). In the study, 18 patients were enrolled in the “puremove” subtype (the average frequency of the five triggers/factors “sudden voluntary actions”, “speed change”, “emotional factors”, “intention action”, and “fatigue” are: 100.00%, 83.33%, 0.00%, 0.00%, 0.00%), while 80 patients were classified into the “non‐puremove” subtype (the average frequency of the five triggers/factors “sudden voluntary actions”, “speed change”, “emotional factors”, “intention action”, and “fatigue” are: 100.00%, 77.50%, 73.75%, 73.75%, 41.25%).

To explore whether different subtypes of PKD have different patterns of neural oscillatory activity, both theta PSD and high‐gamma PEC features were compared in the two subtypes versus HCs. Again, we excluded med‐on state patients’ EEG data (eye‐open: “puremove” *n* = 12, “non‐puremove” *n* = 51; eye‐close: “puremove” *n* = 13, “non‐puremove” *n* = 42). Compared with the “puremove” subtype, the “non‐puremove” subtype has more regions showing significantly increased theta oscillation than HCs (Figure 4b; Figure S6a, Supporting Information). In the eye‐close paradigm, the “puremove” subtype only increased at the right inferior of temporal and left Caudate. In contrast, the “non‐puremove” subtype increased at 20 regions, mainly from CB and SC groups. The results of the eye‐open paradigm were similar and more significant between the two subtypes. These results indicated more engagement of theta oscillation in patients with emotional aggravating factors.

On the other hand, both patients of the “puremove” and “non‐puremove” subtypes showed widely decreased high‐gamma FCs than HCs in the eye‐closed paradigm (Figure 4c). The main aberrant FCs were between the SMN groups and DMN, DAN, FPN, LN, and SC, or within the DAN and SMN groups. However, no similar results were found in the eyes‐open paradigm (Figure S6b, Supporting Information). These results indicated that high‐gamma oscillation might be more associated with PKD patients with motor‐related triggers alone.

### 3.6 Performance of Prediction Models for Identifying Remission State

**Figure 5:**
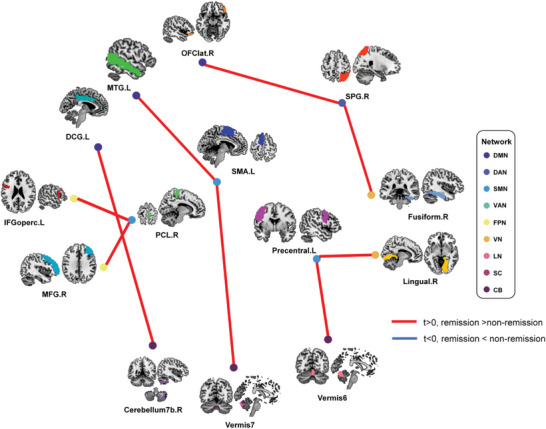
The Top nine significant differences in high‐gamma oscillation FC between remission and non‐remission PKD patients. DCG: Middle Cingulate & Paracingulate gyri; IFGoperc: Inferior Frontal Gyrus, opercular part; MFG: Middle Frontal Gyrus; MTG: Middle Temporal Gyrus; PCL: Paracentral Lobule; SMA: Supplementary Motor Area; SPG: Superior Parietal Gyrus.

In order to explore whether neural markers could assist in clinical prognosis, a CPM was established based on the PSD and PEC features between remission and non‐remission PKD groups. Only features with *p*‐values ˂0.3 were selected as input since they got the best performance on both dataset 1 and dataset 2. The oscillatory activity of several regions from the cerebellum at low‐gamma and high‐gamma oscillation remarkably differed between remission and non‐remission PKD patients (Figure S7, Supporting Information). The most significant differences in FC (**Figure** **5**, Supporting Information) were all found in high‐gamma oscillation and from SMN, CB, DMN, FPN, VN, and DAN. Moreover, three regions in SMN (PCL, SMA, Precentral) and one in DAN (SPG) were the critical nodes and connected to other regions. More details about the difference between remission and non‐remission PKD groups were shown in Figures S7 and S8 (Supporting Information) in both the eye‐open and eye‐close paradigms.

**Figure 6:**
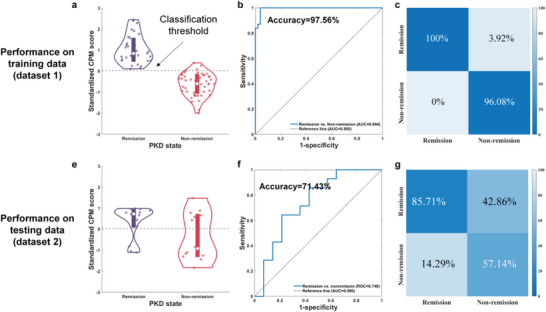
Performance of CPM for identification remission and non‐remission patients. a) The distribution of CPM scores of remission and non‐remission PKD patients in training data (dataset 1). b,c) Receiver operating characteristic (ROC) and sensitivity and specificity of classification performance on training data. The same CPM was used on dataset 2 for validation, and its performance was shown in (d–f).

The performances of CPMs for identifying PKD with or without remission in dataset 1 and dataset 2 are shown in **Figure** **6**. The scores of CPM distribution varied significantly between the two subtypes in dataset 1. The accuracy of the CPM was 97.56%, and the AUC of ROC was 0.994 with high sensitivity and specificity. The accuracy of the CPM for classification in dataset 2 was 71.43%, and the AUC of the ROC curve was 0.735. The results indicated that this CPM could effectively identify PKD patients with remission with an 85.71 chance but only could correctly identify unremised PKD patients with a 57.14% chance. We believe such differences are mainly due to the more significant heterogeneity of non‐remission patients regarding disease symptoms, medication states, and other factors. We tested the impact of the threshold *p*‐value for feature selection (*p*‐value< [0.001, 0.01, 0.05, 0.1, 0.2, 0.3, 0.4, 0.5]) on the prediction results (Figure S9, Supporting Information). The results showed that all selected thresholds in the training dataset (dataset 1) could effectively distinguish PKD patients with or without remission. When the *p*‐value was over 0.01, the CPM model can also effectively distinguish the remission state in the test dataset (dataset 2) with an AUC of ROC larger than 0.5.

## 4 Discussion

The current study first established a clinically relevant electroencephalography profile of PKD. According to our results, the neural activity of PKD is widely changed at oscillatory activity and functional connectivity in all frequency bands. The most remarkable changes are increased theta oscillation activity and decreased high‐gamma FCs, which are not only related to disease progression but also specifically responsible for different subtypes of PKD. Furthermore, based on these notable findings, a powerful neurobiomarker has been formed to establish a clinically translational model to evaluate the prognosis of PKD.

### 4.1 The Increased Theta Oscillation Is a Crucial Feature of PKD and May be Related to the Abnormal Psychological State of PKD

Compared to HCs, approximately half of the brain regions showed significantly higher activity at theta oscillation in eye‐open and eye‐closed paradigms. The most involved regions are SMN, DAN, SN, and CB. However, aberrant activity would be dismissed with the relief of PKD symptoms. These results indicate that enhanced theta oscillation is a crucial feature of PKD. Previous studies showed that abnormal theta activities in the SN and DAN might be involved in abnormal cognition and psychological states, and the primary physiological function of theta oscillation is memory processing, especially in the cortico‐hippocampal circuit.^[^ ^30^, ^31^ ^]^ Clinical studies have shown that increased theta oscillation is a typical phenomenon after acute brain lesions.^[^ ^32^ ^]^ However, its role in neural disorders is controversial, as the enhanced presence of theta oscillation may suggest network disassembly, and it might also be a signal promoting plasticity in the context of the reorganization of the neural network.^[^ ^33^ ^]^ This controversy was also found in this study. As shown in Figure S4b (Supporting Information), significant theta FCs mainly increased in the PKD patients compared with HCs, except for FC from the cerebellum to other regions. Therefore, we assume that the abnormal theta oscillation in the cerebellum of PKD might be related to the impaired information transmission between the cerebellum and other brain regions, leading to impaired motor integration function.^[^ ^10^ ^]^ Combined with the results shown in Figure 4b, only the “non‐puremove” PKD subtype showed wildly increased theta activities in brain regions, we presume that increased theta FCs between noncerebellar regions, especially from the SN and DAN, is probably attributed to plasticity in neural network reorganization induced by cognition and psychological change under a chronic disease state.^[^ ^27^ ^]^

### 4.2 Decreased High‐Gamma Functional Connectivity Is Associated Explicitly with Motor Symptoms of PKD

PKD patients experience spontaneous remission with age. In this study, decreased high‐gamma FCs were found only in the “non‐remission” group. Moreover, non‐remission PKD patients have a more significant gap in FC than HCs compared to remission patients. These results indicate that abnormal high‐gamma FCs in PKD are closely related to the severity of motor symptoms. It is known that gamma oscillations originate from GABAergic inhibitory interneurons, which play a crucial role in sensorimotor integration in the cortical‐cortical and thalamocortical circuits.^[^ ^34^, ^35^ ^]^ The impaired connectivity on a high‐gamma oscillation in PKD patients involves a broader range of networks, including the DAN, SMN, VAN, FPN, and VN, which are in charge of our sensory and motor functions and are associated with movement disorders. In addition, as the decreased high‐gamma FC was found both in the “puremove” and “non‐puremove” subtype of PKD, we assumed that the alteration in gamma FCs is consistent with the motor symptoms of PKD. A similar assumption was proposed in one previous MEG study as well.^[^ ^15^ ^]^

### 4.3 PKD Is a Network Disorder Involving Almost All Brain Regions

The remarkable changes in PKD involve almost all brain regions at both the oscillatory activity and FC. This result is consistent with previous imaging studies, which revealed a wide range of brain regions associated with PKD.^[^ ^36^, ^37^ ^]^ Similarly, in other hyperkinetic movement disorders, most networks were affected, mainly the SMN, cerebellar, basal ganglia, cognitive control, and DMN, with different changes for each network.^[^ ^38^ ^]^ Compared to other cortical and subcortical regions, the enrolment of the cerebellum in PKD has been disclosed recently. In this study, the changes in the cerebellum in PKD were more prominent and persistent compared to other regions. Imaging studies on PKD have revealed that patients had decreased inhibition from cerebellar lobule VIII to thalamic relays. In contrast, the connection from cerebellar lobule VI to thalamic relays was less facilitated, and self‐inhibition in cerebellar lobule VI was increased. Meanwhile, connections from the thalamus to the striatum and the primary motor cortex were more facilitated.^[^ ^10^, ^39^ ^]^ Furthermore, *PRRT2*, the primary pathogenic gene of PKD, is highly expressed in the cerebellum, especially in granule cells (GCs), and enriched at the presynaptic membrane in the molecular layer. Cerebellar knockout of *PRRT2* could result in the phenotype of PKD in mice and abnormal excitability of the cerebellum.^[^ ^11^, ^40^ ^]^ The cerebellum integrates sensory and motor information from the cortex, as well as the control of the motor cortex via the thalamus. It participates in motor learning, control, and coordination. Our results supported the cerebellar involvement in PKD and indicated a difference in FC with other hyperkinetic movement disorders. In PKD patients, although decreased FCs in the cerebellum were the main characteristic in the eye‐closed paradigm, other networks were also affected. We assume that this might be attributed to the clinical heterogeneity of PKD, but due to the sample size of the current study, the assumption should be carefully interpreted in further investigations.

Although PKD is considered a benign movement disorder due to the dramatic response to epileptic drugs and its spontaneous remission, it still profoundly impacts patients' quality of life, both physically and psychologically. To date, there are no tools or methods to predict the duration of the disease or evaluate the state of the disorder, which could provide certainty and preparation for patients in their daily lives. Therefore, we attempted to set up a prediction model based on our results. In our CPM model, features from oscillatory activities and FCs showed promising performance on sensitivity and specificity to identify PKD patients with remission. Further validation of the model by external data also showed satisfying performance. Therefore, features from hd‐EEG might be considered translatable neural biomarkers to evaluate the state of the disease and predict the prognosis of PKD. It will help clinicians in the future to create a fast and inexpensive diagnosis assistant system.

### 4.4 Limitations

There are still some limitations in the present study. First, although the current dataset is larger than the published PKD electrophysiological studies, the number of some subtypes is still small, limited by their relatively low natural incidence. In addition, only the resting‐state data were recorded and analyzed, and the results could not show the whole picture of the mechanism of PKD. In the following investigation, we will proceed with the task hd‐EEG, including kinesigenic or emotional triggers/factors, to further understand the abnormal brain activity of PKD. Third, it should be noted that the precision of source estimation in the present study was limited by choosing the average brain template for source localization rather than the individual brain model. However, without collecting an MRI of every participant to estimate individual brain models, the experiment became more efficient, economical, and practical. In the following research, we will apply other methods, such as EMG or MRI, to provide higher‐precision localization of brain regions, such as subregions of the thalamus. Moreover, an MRI would be collected when applying the remission state prediction model for clinical diagnosis.

## 5 Conclusion

In conclusion, our work characterized the whole brain profile of PKD and discovered the key brain regions, significant neural oscillations, and unique functional activity patterns of PKD, which have never been investigated in previous research. The results not only provide a novel reference for the mechanism of PKD as well as a translational and practical tool of clinical evaluation but also exhibit the theoretical evidence and therapeutic targets for further physical treatment in clinical practice.

## Conflict of Interest

The authors declare no conflict of interest.

## Supporting information

Supporting Information

## Data Availability

Research data are not shared.
