# Supplementary material for: An Electroencephalography Profile of Paroxysmal Kinesigenic Dyskinesia
Source: Adv Sci (Weinh). 2024 Jan 16;11(12):2306321. doi: 10.1002/advs.202306321 (PMC10966565; doi:10.1002/advs.202306321)
Supplement: Supplementary file 1 — Supporting Information [file ADVS-11-2306321-s001.pdf]

ADVANCED SCIENCE
Open Access

Supporting Information

for *Adv. Sci.*, DOI 10.1002/advs.202306321

An Electroencephalography Profile of Paroxysmal Kinesigenic Dyskinesia

Huichun Luo, Xiaojun Huang, Ziyi Li, Wotu Tian, Kan Fang, Taotao Liu, Shige Wang, Beisha Tang, Ji Hu, Ti-Fei Yuan and Li Cao**

Power envelope connectivity (PEC)

Here, we provide additional information on how to estimate power-envelope connectivity from two brain regions' signals.

A. Extraction envelope of signal

Before performing PEC, the envelope signal of a specific frequency band needs to be extracted using a complex Morlet wavelet. The specific calculation process includes the complex Morlet wavelet transform of the original signal, taking different center frequencies and different scales to calculate the wavelet coefficients of the specified frequency bands, and then according to the signal demodulation principle in the envelope detection technique, the envelope component in the wavelet coefficients can be derived.

B. Orthogonalizing two signals

The key step of the analysis approach is to orthogonalize two signals before deriving their power envelopes for correlation analysis. This procedure ensures that the signals do not share the trivial co-variability in power due to measuring the same sources while preserving co-variation related to measuring different sources.

After the envelope signals are obtained using complex Morlet wavelets, orthogonalization of the envelope signals is calculated as follows (Figure S1). The envelope signals X(t,f) and Y(t,f) in the frequency band of interest f are obtained by doing complex Morlet wavelet transform on the time domain signals x(t) and y(t) from the two brain regions, respectively.

Then, the part of a complex time series Y that can instantaneous and linearly be predicted from X, i.e. Y||X, is:

$$Y_{||X}(t,f) = a_{X,Y}(f,T)X(t,f) = real\left(\frac{\sum_{t'\in T} X(t',f)Y(t',f)^*}{\sum_{t'\in T} X(t',f)X(t',f)^*}\right)X(t,f)$$

Where $a_{X,Y}$ is the regression coefficient that describes the instantaneous linear relation between X and Y that is estimated from data in the time interval T, * is the complex conjugate, and real( ) is the real part of a complex number. The signal Y orthogonalized to the signal X, i.e. $Y_{\perp X}(t,f)$, can be derived by subtracting the parallel signal component:

$$Y_{\perp X}(t,f) = Y(t,f) - Y_{||X}(t,f)$$

$$Y_{\perp X}(t,f) = Y(t,f) - real\left(\frac{X(t,f)Y(t,f)^*}{|X(t,f)|^2}\right)X(t,f) = Y(t,f) - real\left(\frac{X(t,f)}{|X(t,f)|}Y(t,f)^*\right)\frac{X(t,f)}{|X(t,f)|}$$

$$Y_{\perp X}(t,f) = imag\left(Y(t,f)\frac{X(t,f)^*}{|X(t,f)|}\right)\hat{e}_{\perp X}(t,f)$$

$$\hat{e}_{\perp X}(t,f) = \frac{iX(t,f)}{|X(t,f)|}$$

$\hat{e}_{\perp X}(t,f)$is the unit vector perpendicular to X.

C. Pearson correlation between Orthogonalized two signals

The PEC is the results in a positive number $|Y \perp X(t,f)|$ which is then squared and log-transformed, and correlated with $|X(t,f)|$ by Pearson correlation test.

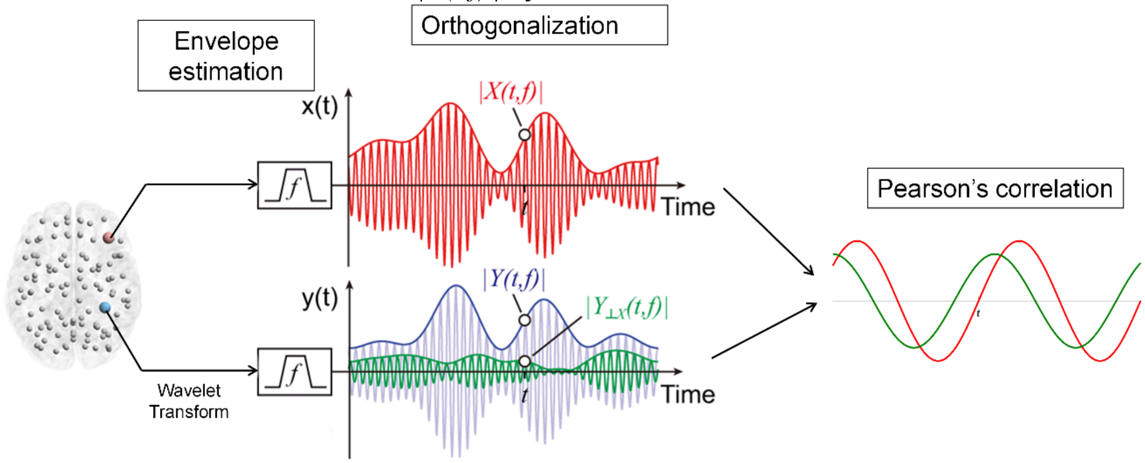

Figure S1. Three steps for PEC calculation. This figure references the paper "Hipp et al., 2012, Nature Neuroscience" and adjusts for this study.

Connectome-based predictive modeling

For each subject, inputs to CPM are a feature matrix and group label of PKD state. Step 1: Feature matrices on six oscillations are from oscillation's power of regions and function connectivity. Step 2: The input data includes a training set (dataset 1) and a testing set (dataset 2). Step 3: Across all subjects in the training set, each edge in the feature matrices is related to the group label using the Student's test. Step 4: After the Student's test, the most important features are selected for further analysis. Step 5: The most important edges are summed into a single subject CPM score for each subject. Step 6: A predictive model is built based on logistic regression between CPM scores and group labels. Step 7: CPM scores are calculated for each subject in the testing set. This score is then input into the predictive model. The resulting value is the predicted PKD state for the current test subject.

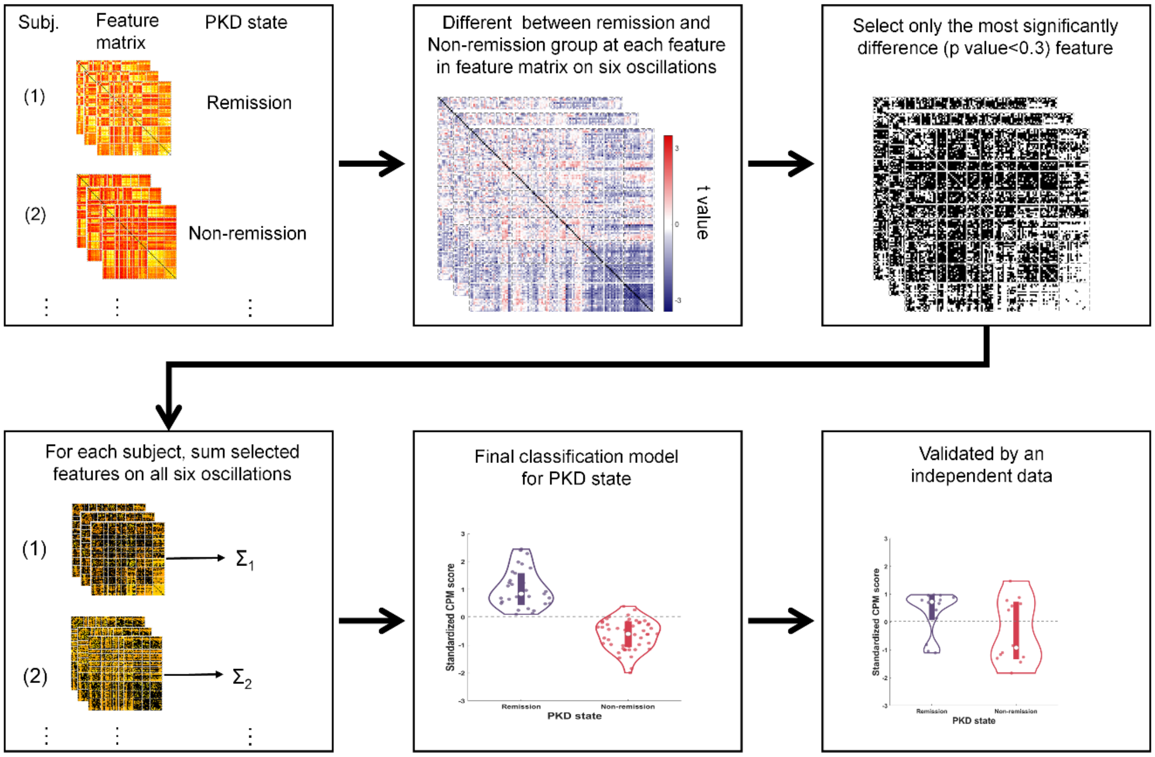

Figure S2. Schematic of CPM for classification remission and non-remission state of PKD patients. This figure references the paper "Shen et al., 2017, Nature Protocols" and adjusts for this study.

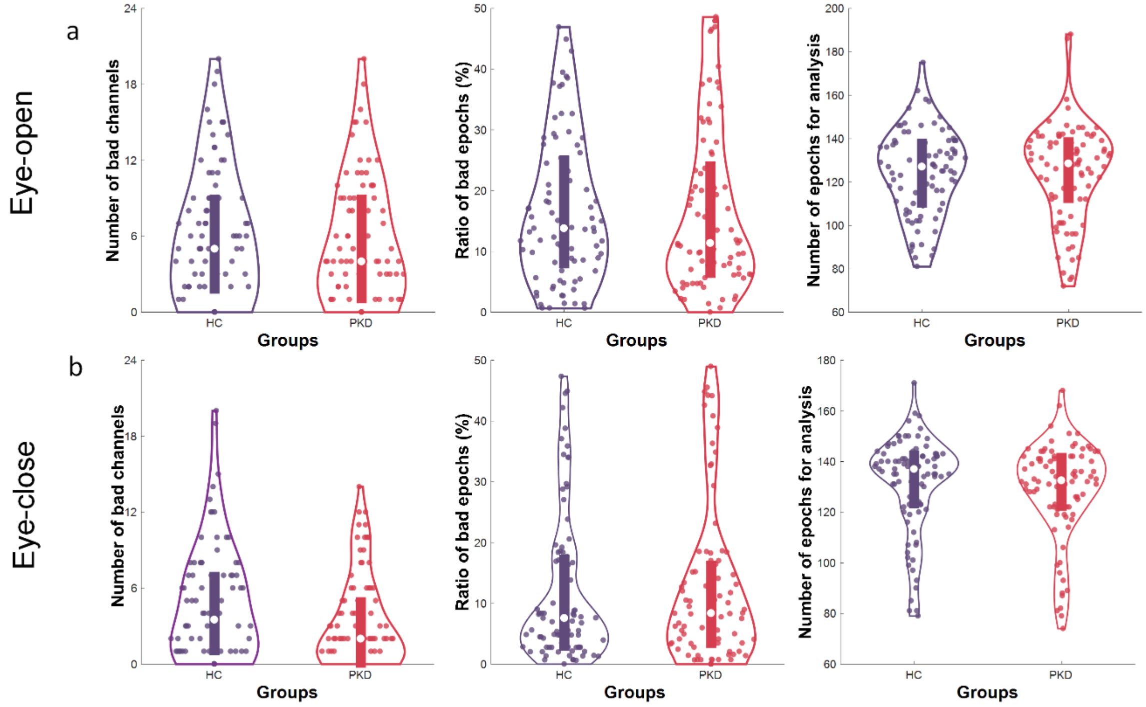

Figure S3. The number of bad channels, the ratio of bad epochs, and the number of epochs for analysis of eye-open (a) and eye-close (b) EEG data of subjects in HC and PKD groups.

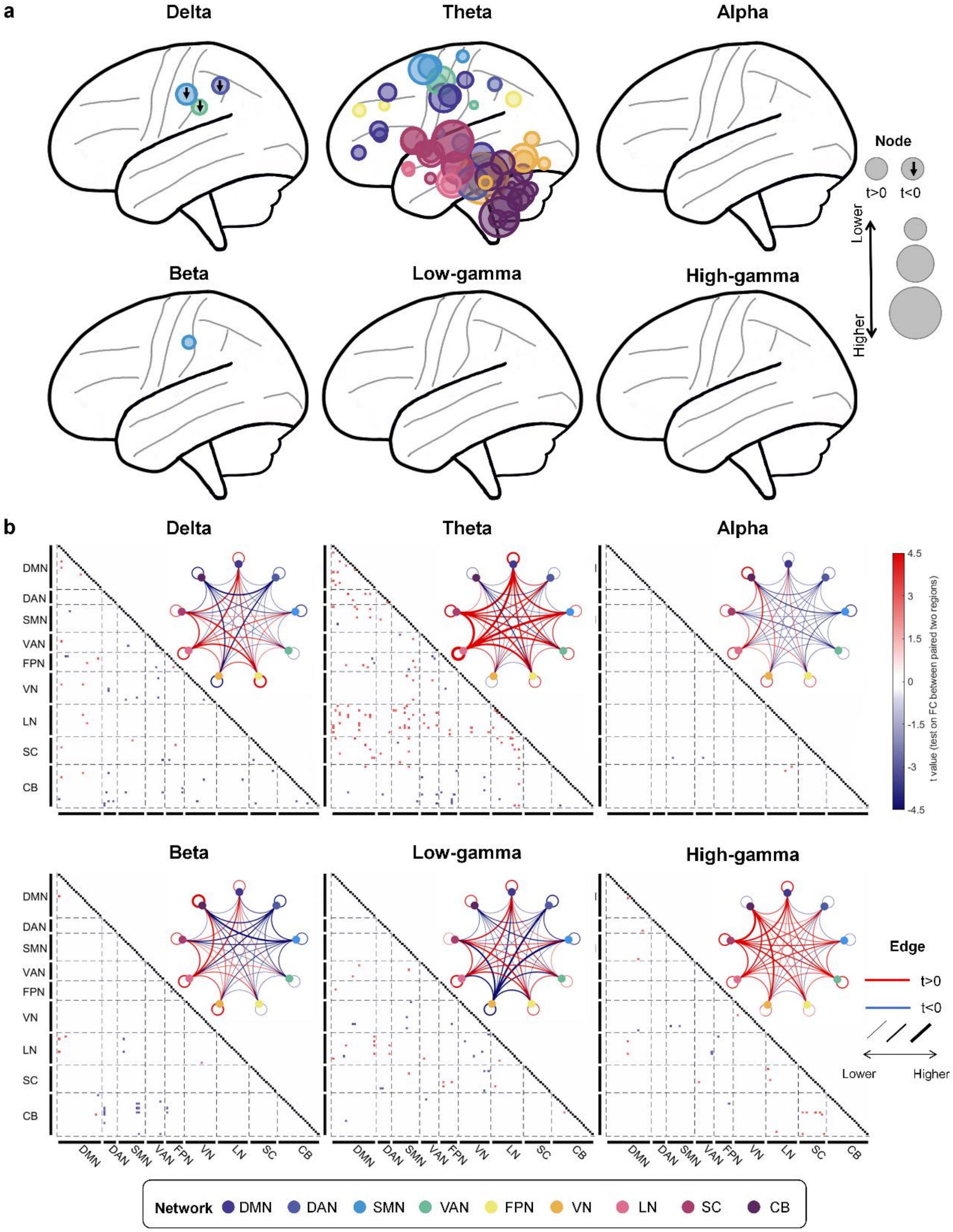

Figure S4. The discrepancy oscillatory activity pattern between PKD and HC on the eye-open paradigm. (a) Visualization of oscillatory activity difference (PKD vs. HC) patterns on the brain. The node size at each significant region (corrected p-value <0.05 after FDR correction) represents its absolute t-value, the large the size, the more significant it becomes. A downward arrow on the node indicates that PKD activity is significantly lower than HC (t-value <0), and the absence of an arrow indicates that PKD activity is significantly higher than HC (t-value>0). **(b)** Functional connectivity difference between PKD and HC. Squares in the triangular matrix are functional connectivity (FC) of paired brain regions, and only significant FCs are drawn (p<0.05 after FDR correction). The black squares indicate that the diagonal elements in the FC matrices are non-meaningful for our analysis. We averaged the t-values for each network and displayed them with a circular plot. The circle color represents different networks; the lines between or within the circle are the averaged t-value. The blue line indicates that the t-value is greater than 0, while the red line indicates that the t-value is less than 0, and the thicker the line, the greater the absolute value of t.

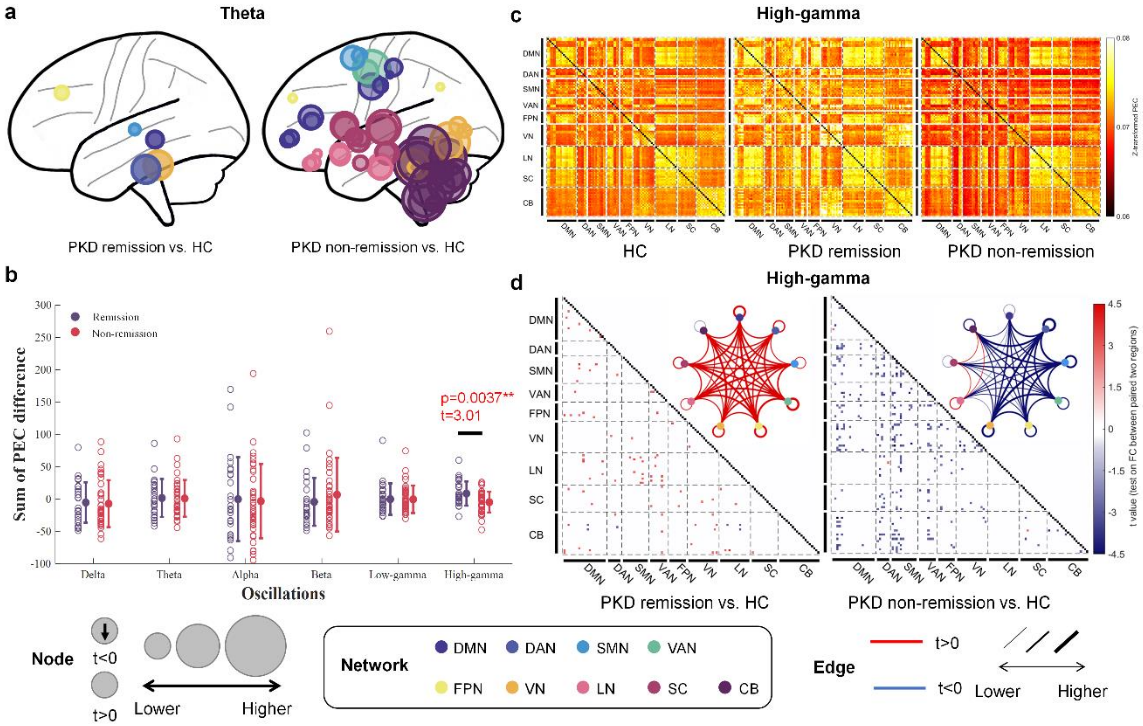

Figure S5. The discrepancy oscillatory activity pattern of remission and non-remission PKD patients on the eye-open paradigm. **(a)** Regions with significantly increased theta activity between remission or non-remission PKD patients and HCs. **(b)** Mean high-gamma oscillation FC matrices of all HCs, remission and non-remission PKD patients. **(c)** The sum of PEC differences between remission or non-remission PKD patients and HCs. The non-remission PKD patients were significantly higher than the remission PKD patients at the PEC on high-gamma oscillation. **(d)** High-gamma oscillation FC difference between remission or non-remission PKD patients with HC.The black squares in (d) indicate that the diagonal elements in the FC matrices are non-meaningful for our analysis.

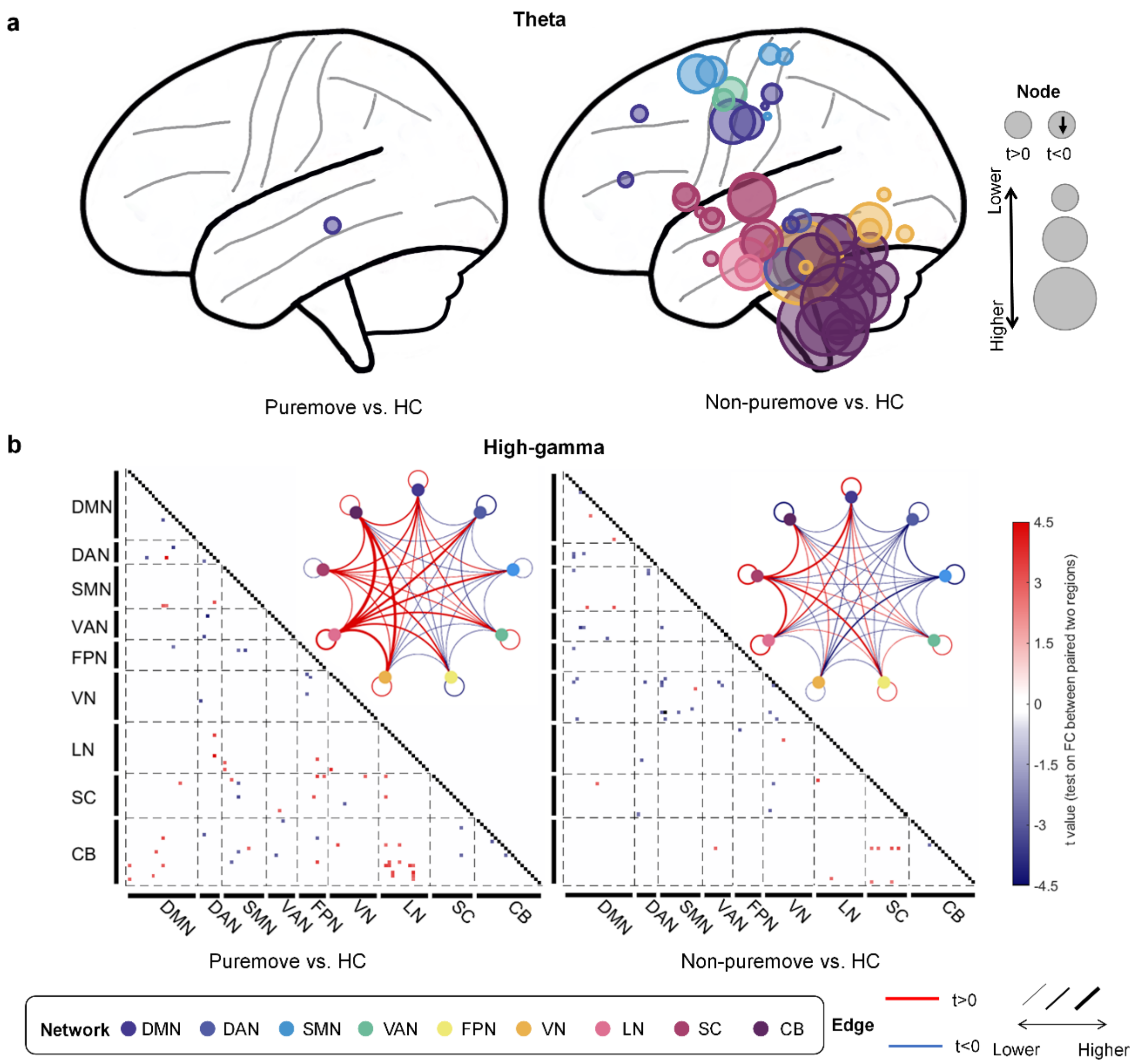

Figure S6. The discrepancy oscillatory activity pattern of two subtypes of PKD: "Puremove" and "Non-puremove" on the eye-open paradigm. (a) Regions with significantly increased theta activity between the two subtypes and HC. **(b)** High-gamma oscillation FC difference between the two subtypes of PKD and HC.

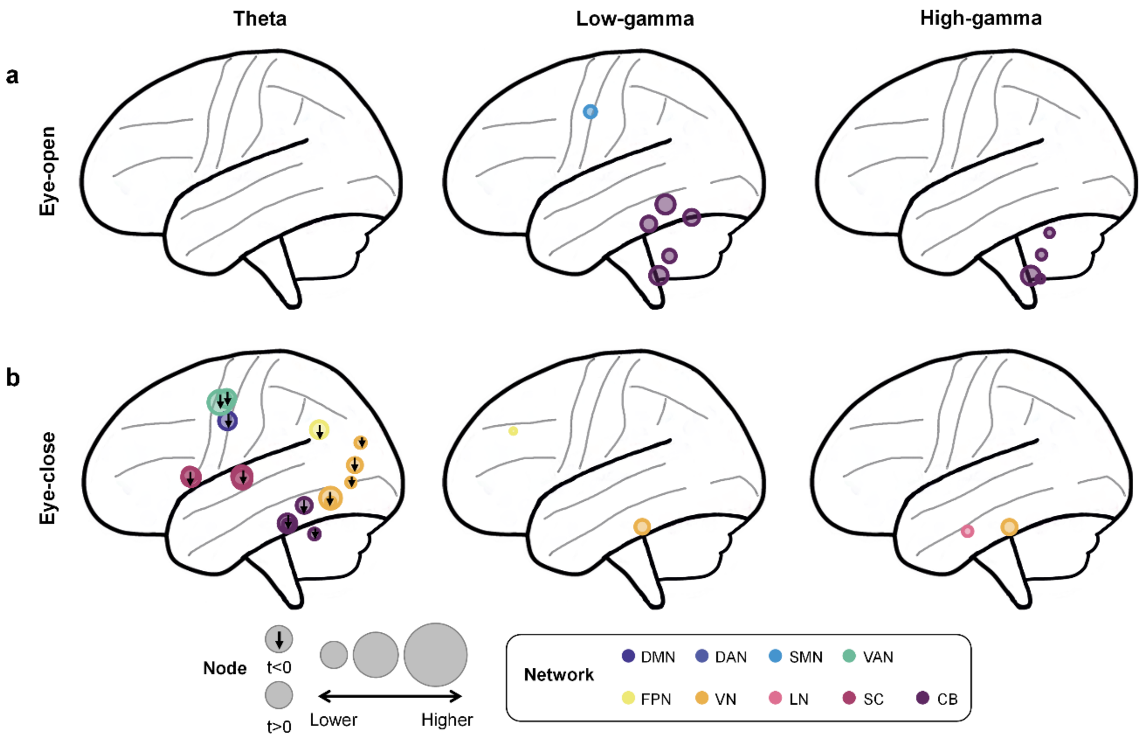

Figure S7. Oscillatory activity difference between remission and non-remission PKD patients. Visualization of oscillatory activity difference (remission vs. non-remission) patterns on the brain: (A) on eye-open paradigm and (B) on eye-close paradigm. Only the theta, low-gamma, and high-gamma oscillation results are shown because there is no significant difference in other oscillatory activity. The node size at each significant region (corrected p-value <0.05 after FDR correction) represents its absolute t-value, the large the size, the more significant it becomes. A downward arrow on the node indicates that oscillatory activity of remission is significantly lower than non-remission (t-value <0), and the node without an arrow indicates that remission is significantly higher than non-remission (t-value>0).

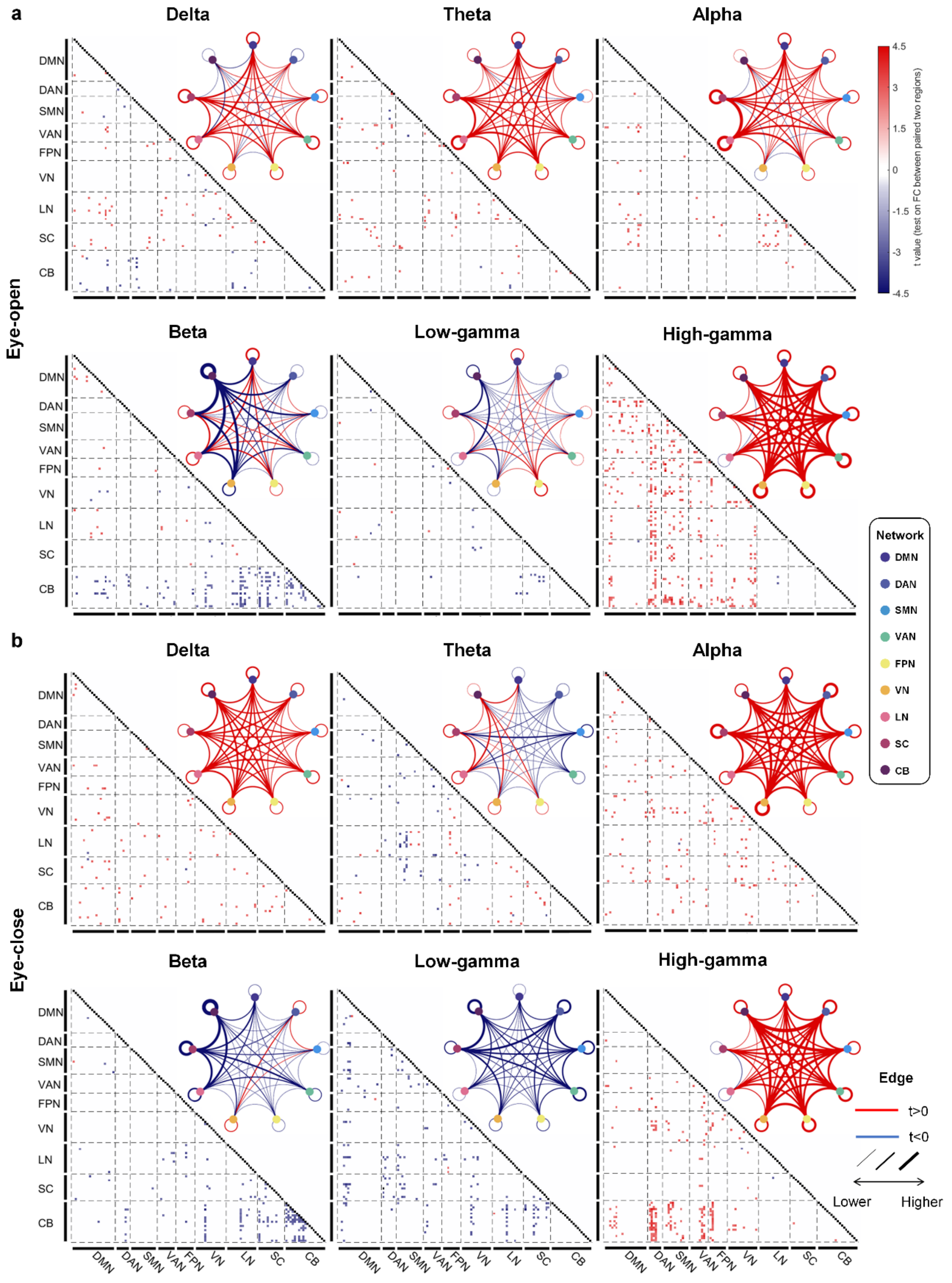

Figure S8. Functional connectivity difference between remission and non-remission PKD patients. (A) Eye-open paradigm and (B) eye-close paradigm. Squares in the triangular matrix are functional connectivity (FC) of paired brain regions, and only significant FCs are drawn ($p<0.05$ after FDR

correction). The black squares indicate that the diagonal elements in the FC matrices are non-meaningful for our analysis. We averaged the t-values for each network and displayed them with a circular plot. The circle color represents different networks; the lines between or within the circle are the averaged t-value. The blue line indicates that the t-value is greater than 0, while the red line indicates that the t-value is less than 0, and the thicker the line, the greater the absolute value of t.

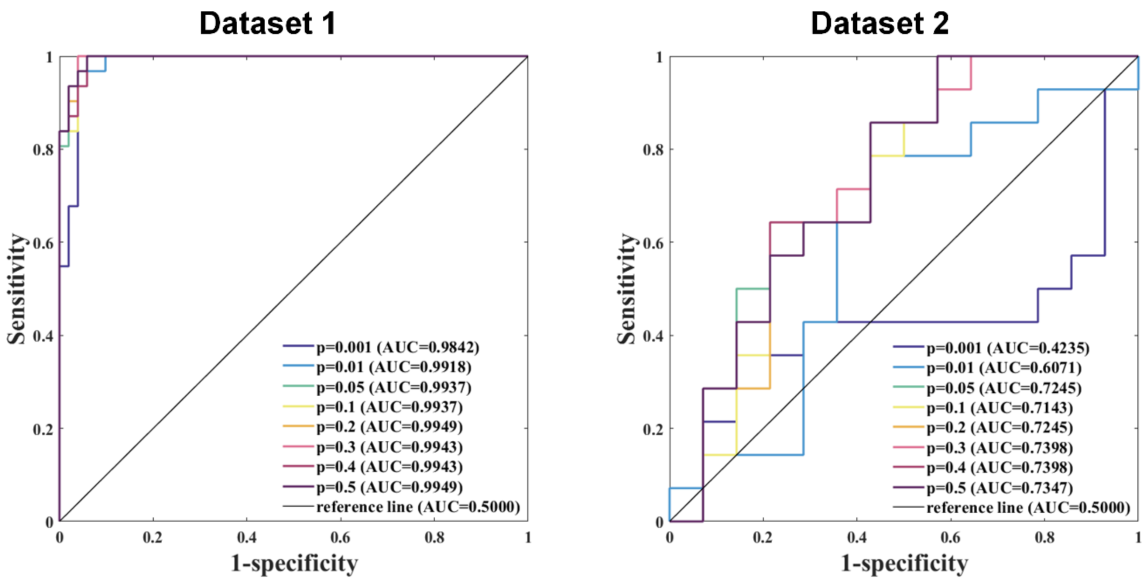

Figure S9. The performance (ROC) of CPM for identification remission and non-remission patients based on different thresholds of p-value on both dataset 1 and dataset 2.
